# Supraglottic Airway Devices with Vision Guided Systems: Third Generation of Supraglottic Airway Devices

**DOI:** 10.3390/jcm12165197

**Published:** 2023-08-09

**Authors:** Caridad G. Castillo-Monzón, Tomasz Gaszyński, Hugo A. Marroquín-Valz, Javier Orozco-Montes, Pawel Ratajczyk

**Affiliations:** 1 Service of Anaesthesiology, Reanimation and Pain Therapy, University General Hospital of Cartagena, 30202 Murcia, Spain; javi_om@hotmail.com; 2 Department of Anaesthesiology and Intensive Therapy, Medical University of Lodz, 90-154 Lodz, Poland; pawel.ratajczyk@umed.lodz.pl; 3 Emergency Medicine University General Hospital of Cartagena, 30202 Murcia, Spain; hamvalz@hotmail.com

**Keywords:** airway management, airway devices, laryngeal mask airway, supraglottic airway devices, video laryngeal mask system, complications

## Abstract

Supraglottic airway devices are currently widely used for airway management both for anaesthesia and emergency medicine. First-generation SADs only had a ventilation channel and did not provide protection from possible aspiration of gastric content if regurgitation occurred. Second-generation SADs are equipped with a gastric channel to allow the insertion of a gastric catheter and suctioning of gastric content. Additionally, the seal was improved by a change in the shape of the cuff. Some second-generation SADs were also designed to allow for intubation through the lumen using fiberscopes. Although the safety and efficacy of use of SADs are very high, there are still some issues in terms of providing an adequate seal and protection from possible complications related to misplacement of SAD. New SADs which allow users to choose the insertion scope and control the position of SAD can overcome those problems. Additionally, the Video Laryngeal Mask Airway may serve as an endotracheal intubation device, offering a good alternative to fibre-optic intubation through second-generation SADs. In this narrative review, we provide knowledge of the use of video laryngeal mask airways and the possible advantages of introducing them into daily clinical practice.

## 1. Introduction

One of the first supraglottic airway devices (SADs) on the market was the Laryngeal Mask Airway (LMA), a term coined over forty years ago by its inventor, Dr. Archie Brain, who revolutionised airway management. He dedicated his life to teaching people to use the device and making it safer and friendlier. In his first publication on the LMA Classic, he advised evaluating with a laryngoscope if the device was not working properly [1].

We have witnessed the continuous development of SADs, their improvement and the emergence of new models for adult and paediatric populations. There are studies that explore their use in high-risk populations, although their use is considered controversial in patients at risk of aspiration [2,3,4,5,6]. SADs have been incorporated in anaesthesiological management for different types of operations and in different patient positions [7,8,9]. They are used in other areas such as emergency departments, intensive care units and during rescue by non-medical staff in the pre-hospital environment [10].

The ASA 2022 and DAS 2015 Difficult Airway Algorithms include SADs as fundamental weapons in airway management. They are the “gold standard” treatment in the event of an inability to ventilate and oxygenate the patient.

The possible uses of SADs in difficult airway management are [11,12,13]:

1. As the device of choice after failure of face mask ventilation.
2. As an alternative approach to airway management after failed intubation as a ventilation device/intubation channel.
3. To attempt fibre-optic-assisted (not blind) intubation via SAD.
4. As an airway rescue device in non-ventilatory, non-intubatable situations, prior to the establishment of a surgical airway.

In the United Kingdom, SADs are used in 56% of general anaesthesia procedures [14] and it is possible that 150 million of these devices are used worldwide each year [15].

SADs are generally forgiving devices because even suboptimally positioned SADs still can provide adequate ventilation for patients during short procedures [16]. This statement has been proven since 1991 by radiological studies [17,18] and by fiber-optic evaluation performed after LMA insertion in patients who were relaxed [19].

The most widely used classification of SADs is that published by Timmemann et al. in 2011 [20]. He divides them into first-generation devices, which have only a breathing tube, and second-generation devices, which have a breathing tube and a gastric channel to protect against aspiration, resulting in better sealing pressures. Van Zunderck proposed classifying third-generation SADs as those with built-in video [21].

The evolution in this field is no different from what has happened in other areas of the specialty. Before the introduction of ultrasound, central venous catheters were placed and nerve blocks were performed using anatomical landmarks; today, procedures are performed with ultrasound guidance, so procedures are safer, blocks are successful, and the volume of local anaesthetics used can be reduced. The development of new video-assisted devices is a qualitative leap in airway management.

The optimal anatomical position of a SAD should be as follows [22]:

- The distal tip of the SAD must rest on and block the upper oesophageal sphincter.
- The cuff occupies the entire hypopharynx and is positioned behind the cricoid cartilage, anterior to the second to seventh cervical vertebrae.
- The opening of the SAD opposes the glottis.
- The sides of the cuff lie in the pyriform fossae.
- The epiglottis is located external to the device, aligned with the proximal part of the laryngeal mask (The epiglottis determines the correct placement of any SAD.).
- The superior border of the mask lies at the base of the tongue.
- Two appropriate seals must be produced for the respiratory and digestive tract.

The success of a LMA that is well placed on the first attempt can be 75.8% [16], 88% [23], 95% [24] and 95.5% [25], depending on the experience of the anaesthesiologist.

Several techniques for LMA insertion have been proposed [26,27] and laryngoscope-guided insertion is one of them. It has been found to be a useful technique for LMA insertion [23,28]. With the guidance of a direct laryngoscope, it is possible to align the LMA with the laryngeal skeleton and raise the epiglottis so the epiglottis does not block the vocal cords. Campbell et al. [28] used fibre-optic examination to compare the traditional blind insertion technique with direct visual placement using a laryngoscope. They reported that appropriate positioning of the LMA had been achieved in 91.5% of patients in the direct visual placement group, compared with 42% in the blind insertion group.

Although Brain recommended extension of the head and flexion of the neck (sniffing position) for the standard insertion method of the LMA [19], at present it is not clear whether the position of the head and neck affects the placement of the LMA as it does in tracheal intubation. The sniffing position has two components: neck flexion, achieved with head elevation at C5–C6, and head extension. Head elevation as a single component is not sufficient to achieve an adequate sniffing position but is necessary to achieve maximum extension of the occipito–atlanto–axial complex. When extension is limited, poor laryngeal visualisation makes it difficult to intubate the patient [29].

It should be borne in mind that the head elevation in the sniffing position needs to be reproduced because it may vary from one individual to another depending on the length of the neck, the anterior posterior diameter of the thorax and the size and shape of the head in relation to the thorax. Therefore, the height of the pillow to be used depends on the patient’s anatomy and there is no standard size for all circumstances. In the sniffing position, the horizontal alignment of the external auditory meatus with the sternal recess should be verified in a profile view in both normal weight and obese adults [30,31]. In clinical practice, the line joining these two points is often used as an appropriate sniffing position in the non-obese patient [30]. And it represents the ramp position in the patient with BMI ≥ 40 [32,33].

Most investigators elevate the head and do not consider neck flexion for LMA placement, using standard pillows when each patient’s anatomy is different.

However, little is known about the association of the head elevation degree and LMA insertion [24]. Changes in the head and neck position may significantly affect the performance of SAD by altering the pharyngeal structure [34].

High head elevation of 14 cm increased the first attempt success rate of LMA Supreme insertion (96.4%) compared with conventional head elevation of 7 cm height [24].

Studies have found no difference comparing three positions, neutral and maximal flexion/extension, with more than 75% of badly positioned devices evaluated with fibre-optics [35].

Okuda et al. [36] found that at different head and neck positions, ventilation was optimal, although the epiglottis was occupying a significant space within the aperture of the LMA.

A systematic review and meta-analysis of the influence of head and neck position on the performance of SAD showed that the flexed neck position significantly improved airway sealing but adversely affected ventilation and the fibre-optic view for most SADs [34].

Factors which contributed to the failure of their correct placement [29,37]:

- Lack of experience of the operator.
- The technique used.
- Inadequate plane of anaesthesia.
- Inadequate choice of SAD size.
- Patient anatomy: edentulous; patients with ogival palate or a history of temporomandibular joint dysfunction.

These devices are not used in patients with cervical spine instability, high risk of bronchoaspiration, supraglottic pathology, pharyngolaryngeal pathology, limited mouth opening (the introduction of the LMA requires an opening greater than 1.5 cm), morbid obesity and obstructive sleep apnoea, in the latter two because the airway collapses when neuromuscular relaxation is lost [29]. 

## 2. We Should No Longer Use SADs without Vision-Guided Systems

Until recently, the placement of an SAD was a blind procedure and contrary to the belief that its placement led to proper positioning, the literature suggests that despite the clinical tests to verify proper placement, in 50 to 80% of cases its placement is deficient [38,39]. Blind insertion of a SAD can lead to errors. Keller et al. observed an optimal position of SAD in only 29% of all insertions and 79% were badly positioned [40]. Fullekrug et al. [41] found that using blind insertion, 87 out of 100 patients had some encroachment of the glottic opening and 13 had more than 51% narrowing. Campbell et al. [28] found that the LMA placed blindly was initially placed in its best position in 16 out of 38 (42%) of the patients.

Van Zundert et al. [42] found, using video laryngoscopy, that 71% of SADs were initially badly positioned and could be corrected by applying a jaw thrust and lifting the chin. This improves the insertion conditions by raising the epiglottis and increasing the anteroposterior diameter of the pharynx. In 9% of cases, the laryngeal mask had to be replaced because it was too large or too small even though the manufacturer’s recommendations were followed. 

The use of SADs with vision-guided systems prevents and helps to correct suboptimal positions by allowing the diagnosis of the problem, although it is not possible to solve it in all cases. Situations such as a larger laryngeal mask needing to be fitted to raise the epiglottis or if the LMA is too shallow or too deep can be solved.

## 3. Assessment of SADs

With the advent of SADs with video-assisted systems, many of the clinical signs used to assess the correct positioning of these devices will be left behind, such as [16,22,28]:

- Sign at placement: the presence of resistance at the end of insertion.
- Outward movement of the LMA with inflation of the cuff.
- The adverse suprasternal notch tap test (also known as the “Brimacombe bounce”; tapping the suprasternal notch or cricoid cartilage and observing simultaneous movement of a column of lubricant or a soap bubble membrane at the proximal end of the drain tube).

Oropharyngeal airway seal [39,40]: This sign has long been considered a measure of successful placement and adequate performance, with normal oropharyngeal leak pressure values being between 20–25 cm H_2_O. It was even claimed that higher oropharyngeal leak pressure (>25 cm H_2_O or >8 cm H_2_O above the peak inspiratory airway pressure under positive pressure ventilation) ensures adequate ventilation and protection of the airway to reduce the risk of aspiration [43].

The inadequate placement of SADs has been corroborated by clinical, radiological [17,18], magnetic resonance imaging (MRI), computed tomography (CT) [38] and lateral neck X-ray, fibre-optic [28,36,44] and ultrasound evaluations [45,46,47].

Studies using fibre-optic examination, CT or MRI, show that the airway can be functional and clinically acceptable even when the anatomic placement is less than perfect [15,40].

A radiological CT study evaluated the post-mortem placement of SADs and found 14% of devices to be misplaced [38]. CT scans have revealed that the epiglottis is posteriorly deflected against the posterior pharyngeal wall in most (80%) patients [48].

Fibre-optic inspection after LMA placement showed that the epiglottis rests on the outside of the laryngeal mask [22]. Fibre-optic examinations conducted through the LMAs may not reveal the vocal cords 14.3% of the time, even when the device is functioning optimally [16]. Nearly 50% of the time, the tip of the epiglottis may lie within the bowl of the device [17]. Fibre-optic assessment after SAD placement helps to confirm incorrect positioning but does not allow corrective manoeuvres to bring the device into optimal position in real time. It is an invasive method: the patient’s ventilation has to be interrupted to perform it and it may result in contamination of the airway by secretions [20]. Fibre-optic assessment of LMA position performed intraoperatively may be advantageous. In one study, the arytenoids herniated through the mask aperture after one hour of positive pressure ventilation [36].

Ultrasound (US) is a fast, non-invasive, radiation-free method used to assess the airway; it can even help predict difficult laryngoscopy [49] and can detect device rotation. It is a technique that is waiting to be standardised. With US imaging, one can rapidly visualise the tongue, epiglottis, and oesophagus, making it a highly sensitive tool to determine the positioning of SAD [50]. The only requirement is an image that can detect the end of the cuff and show the surrounding structures, as these can be used to confirm correct orientation and contact with the larynx [51].

Studies have found that US seems to be as effective as fibre-optic examination of SAD placement, although it could not detect suboptimal depth of SAD [39,51], as it indicated the need for reinsertion and did not require ventilation to be interrupted. Song et al. performed 12.2% of LMA reinsertions when determining the position of the LMA via ultrasound, finding that a US score predicted the need for reinsertion with sensitivity and specificity of 85.7% and 94.1%, respectively [45]. Ajithan et al. found the composite US score sensitivity and specificity for reinsertion to be 80 and 100% [46]. It has also been evaluated in the paediatric population and it was found that the use of US to verify and relocate LMA placement is effective. Suboptimal LMA placement was detected at a rate of 20.8%; 15.9% were correctable and 4.9% had to be repositioned [47].

US is as accurate as MRI for assessing airway structures [49,52] and is a pending tool for diffusion and use in the airway.

By evaluating two different LMAs with video laryngoscopy, the authors found that corrective manoeuvres were required in virtually all patients to obtain a correct anatomically positioned LMA; 18% had to be size corrected with LMA Supreme and 4% with LMA Protector [39]. An algorithm for SAD assessment with video laryngoscopy has been proposed and using the “insert-detect-correct-as-you-go” technique at the same time allows immediate correction of incorrectly positioned device [22].

But even though the SAD with a video-assisted system is well placed, we will continue to evaluate:

- Appropriate chest rise with each breath.
- Adequate oxygen saturation.
- Capnography to corroborate a good capnography tracing.
- The device pressure.
- Insertion of an orogastric tube through the gastric channel without resistance.

## 4. Incorrect Positioning and Complications

SADs do not provide complete protection against aspiration or regurgitated stomach contents, even if correctly placed [43]. A meta-analysis of the incidence of aspiration associated with LMA use revealed that the risk of pulmonary bronchial aspiration is 0.02% (2 per 10,000 users) [53,54]. Controlled studies have shown that the incidence of silent pharyngeal regurgitation in general anaesthesia with this device can be as high as 5% [55].

To avoid complications, it is essential to ensure that the LMA is correctly positioned in the patient.

**Causes of laryngeal mask bad positioning** [22,56]:

- Incorrect position of the epiglottis [57,58]. The epiglottis represents the most common cause of airway obstruction. Some have found that the epiglottis is deviated backwards in more than 80% of patients after blind insertion [22], while downfolding of the epiglottis occurs in 20–56% of patients [59]. The initial blind insertion resulted in epiglottis downfolding and positioning of epiglottis in the bowl of the device in >75% of cases [39]. It should be taken into consideration that in order not to encounter a high incidence of downward folded epiglottis, it is important to use the right size LMA.
- Hyperinflated/hypoinflated cuff.
- Cuff bent creating airway leaks.
- If the SAD too small or too large, with insertion that is too deep or too shallow, it would produce an inadequate seal.
- Rotation of the SAD cuff in a sagittal plane.

**Inadequately positioned SAD leads to complications**:

- Ventilatory failure, including insufficient tidal volume due to an air leak.
- Airway obstruction.
- Twenty-six times greater likelihood gastric insufflation and subsequent aspiration.

**Complications associated with the use of supraglottic airway devices:** These complications are relatively rare and most of them are not life-threatening [29,38,39].

- Sore throat [54,60] with an incidence of 5.8–34% compared with 14.4% to 53% in association with endotracheal intubation. Limiting cuff pressures may also decrease this incidence.
- Odynophagia [29,55] with an incidence of 7–17%. In endotracheal intubation, the incidence of this complication is 30–49%.
- Injury to the vocal cords. Transient bilateral vocal cord paralysis [61].
- Injury to the epiglottis, arytenoid dislocation [62], uvula [63,64] and pharyngeal pillars due to difficult insertion.
- Nerve injuries: lingual and hypoglossal nerves injury [65], bilateral hypoglossal nerve injury [66], unilateral hypoglossal nerve palsy [67,68], lingual nerve paralysis [69,70], recurrent laryngeal nerve [71].
- Temporomandibular joint dysfunction [72,73]. Insertion manoeuvres such as a jaw thrust may result in temporomandibular joint dysfunction by anteriorly displacing the jaw.

For all these reasons, knowing that bad positions can be corrected by using SADs with video-assisted systems, we can avoid them. By looking at the anatomical structures in real time while a SAD is being placed, we could solve the problem; for example, by applying a thrust of the jaw and lifting the chin. These manoeuvres lift the epiglottis, which is left out of the insertion path, and increase the anteroposterior diameter of the pharynx.

## 5. Supraglottic Airway Devices with Vision Guided Systems Are Here, to Stay 

Third-generation SADs will be the gold standard for safety, allowing us to see the vocal cords, the epiglottis (first seal) and the arytenoids, confirming their proper placement in situ. They can aid with tracheal intubation, allowing us to observe the vocal cord spasm, bronchial aspiration and airway collapse in an obese patient with obstructive sleep apnoea or in a morbidly obese patient in whom the LMA worked perfectly while relaxed. Real-time video-assisted SAD insertion can easily confirm that the chosen device size is adequate and allow immediate correction of any bad positioning due to inadequate sizing, incorrect cuff inflation or poor insertion technique. A limitation could be that it does not provide information on the second seal (distal cuff and oesophagus) [40].

Not all patients will have perfect glottic vision but ventilatory parameters may be adequate; otherwise, endotracheal intubation will be selected if optimal ventilatory parameters are not achieved. 

To assess laryngeal vision, we can use the Cormack–Lehane classification used for direct glottic vision and/or the classification suggested by Brimacombe for assessing fibre-optic glottic vision through the SAD. Neither of these classifications has been validated for indirect glottic vision. 

Brimacombe and Berry [74] introduced a fibre-optic scoring system to standardise the assessment of SAD position. It is most used when [16]:

4: Only vocal cords are visible. This is the optimal position.

3: Vocal cords plus the posterior epiglottis are seen.

2: Vocal cords plus the anterior epiglottis are seen.

1: No vocal cords are visible, but function is adequate.

0: Device failure occurs.

Fibre-optic scores of 2, 3 and 4 are considered anatomically acceptable placements and 1 is considered a poor placement [16]. Different studies find an incidence of score 1 of 9.4% [75,76].

Van Zundert et al. demonstrated that the vision-guided insertion technique could achieve quasi-100% perfect position of a SAD [39].

We have been using blind-fit laryngeal masks for a long time, and the evolution to their use with video may not be easy; the equipment has to be prepared before use and will not be fully accepted if the cost of these disposable devices makes change unfeasible worldwide. Anaesthesiologists may decide not to switch because they are doing well with the blind technique.

## 6. Video Laryngeal Mask (VLMs)

These devices combine the advantages of an integrated videolaryngoscope incorporated into a second-generation SAD.

These are integral and multifunctional supraglottic airway devices with the following features [56,77]:

- The second-generation disposable SAD has separate gastric and ventilation channels allowing functional separation, a silicone cuff and a reinforced distal tip which allows a more compact seal and therefore a higher oropharyngeal sealing pressure.
- It also has a reusable flexible videoscope which is placed into a specially designed blind-end channel terminating in the bowl of the SAD.

Van Zundert et al. [56] described the first two, third-generation devices, those that incorporated a flexible-tipped videoscope:

- The Video Laryngeal Mask VLM^TM^, UE Medical^®^ (SaCoVLM^TM^).
- The SafeLM^®^Video Laryngeal Mask System (SafeLM^TM^VLMS, Magill Medical Technology^®^).

**Figure 1:**
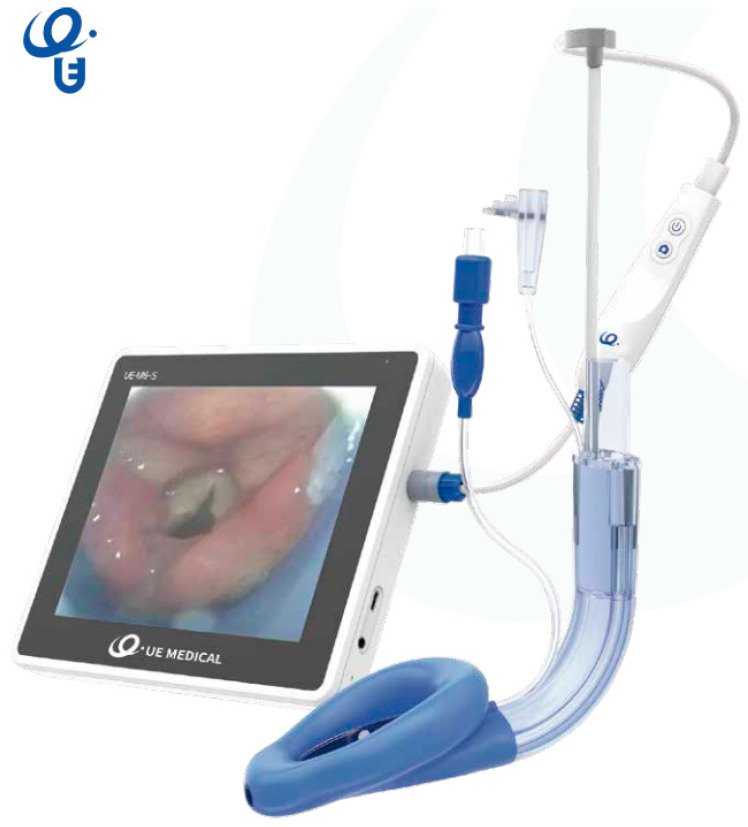
SaCoVLM^TM^ video laryngeal mask airway.

**Figure 2:**
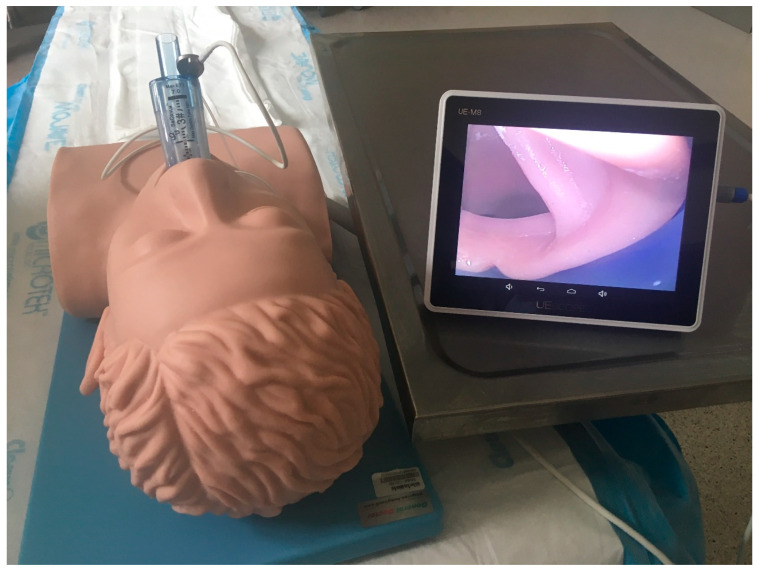
SaCoVLM^TM^ inserted with visualisation of glottis.

**Figure 3:**
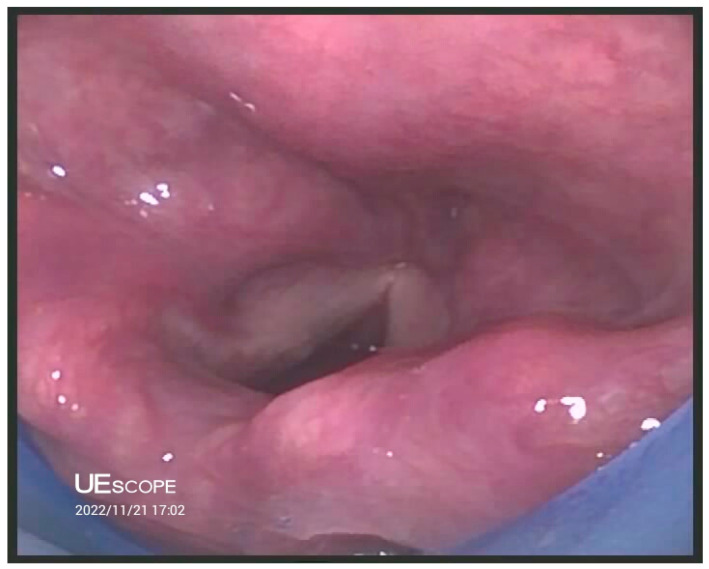
Picture of glottis obtained using SaCoVLM^TM^.

**SaCoVLM^TM^ Video Laryngeal Mask Airway** (Figure 1, Figure 2 and Figure 3).

It consists of two parts: a second-generation disposable SAD with an anatomically curved silicone sleeve and a reusable insulated videoscope with a screen.

This SAD has three channels: a ventilation channel (pre-moulded), a gastric tube channel (16 Fr gastric tube) and a visual tube channel, and it is connected to a separate monitor via a cable. The SaCoVLM^TM^ allows for endotracheal intubation through its lumen under control of vision.

The device has three sizes of laryngeal mask:

- Size 3 is suitable for patients with a body weight between 30 and 50 kg.
- Size 4 is for patients between 50 and 70 kg.
- Size 5 is for patients weighing 70–100 kg. They allow the passage through the ventilation channel of endotracheal tubes of 7.0, 7.5 and 8.0 mm. The size of the gastric tube is 16 French.

Yan et al. [78] tested SaCoVLM™ in airway management for patients under general anaesthesia. They evaluated the device on 100 adult patients scheduled for general anaesthesia. The first pass success ratio of insertion was 95%, and the total success ratio was 96%. The investigators concluded that SaCoVLM™ can visualise partial or whole laryngeal inlets during surgery with a high success rate, a high sealing pressure and smooth gastroesophageal drainage.

Zhi et al. [79] evaluated the application of SaCoVLM™ in 124 anaesthetised children with microtia. They concluded that this is feasible in children, with a high success rate.

SaCoVLM^TM^ can be used for airway management and endotracheal intubation in patients positioned in non-standard (e.g., prone) positions. Intubation in a patient in prone position after unexpected extubation was described [80]. SaCoVLM^TM^ was also successfully used for a predicted difficult intubation in a morbidly obese patient during awake intubation [81]. 

**The SafeLM® Video Laryngeal Mask System (VLMS)** [56,77] 

This is a similar device, but it comes with a camera angle adjusting handle that allows sight under direct vision providing up to a 140° angle (vertical axis) of view of the oropharynx and larynx [18/38]. Its monitor is embedded in the device. The rotation angle of the display screen is 90° along its lateral axis and 270° along its vertical axis. It comes in three sizes (3, 4, and 5), which allows insertion of ETT sizes 6.5, 7.0 and 7.5 mm and gastric tube sizes of 14 Fr for all sizes.

**Figure 4:**
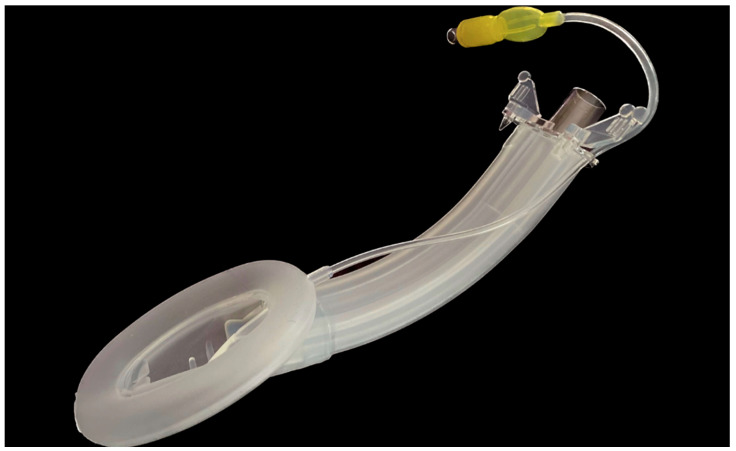
Vision Mask size 4.

**Vision Mask: Video laryngeal mask System.** PMH España. Hospital medical products. Respiratory care solutions (Figure 4).

Vision Mask is the first SAD with continuous vision and five access points. It was developed by Pedro Acha MD., the inventor of Airtraq and Totaltrack. It was presented for the first time in November 2021 at the Anaesthesia Service of the University Hospital Complex of Cartagena, Murcia, Spain, and we have been using it since then.

The Vision Mask complies with the standards set by the European Union in terms of environment, hygiene and safety. It is a disposable silicone mask with integrated vision; it is flexible, has a CPAP option and has two anti-secretion membranes.

This device has three sizes, and the size is selected according to the weight of the patient. (Table 1). 

**Figure 5:**
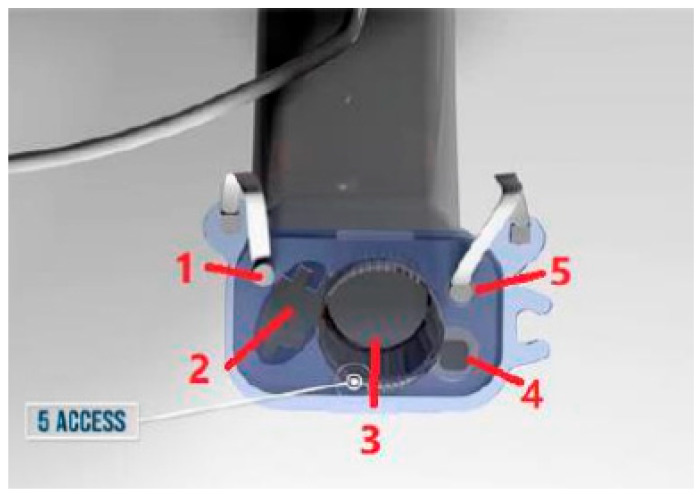
Video system of Vision Mask.

**Figure 6:**
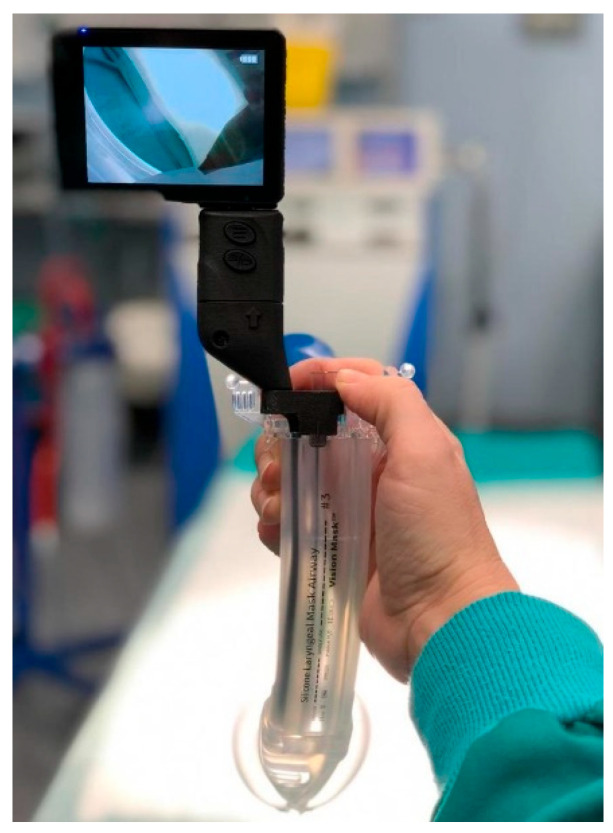
Vision Mask with inserted video system.

**Figure 7:**
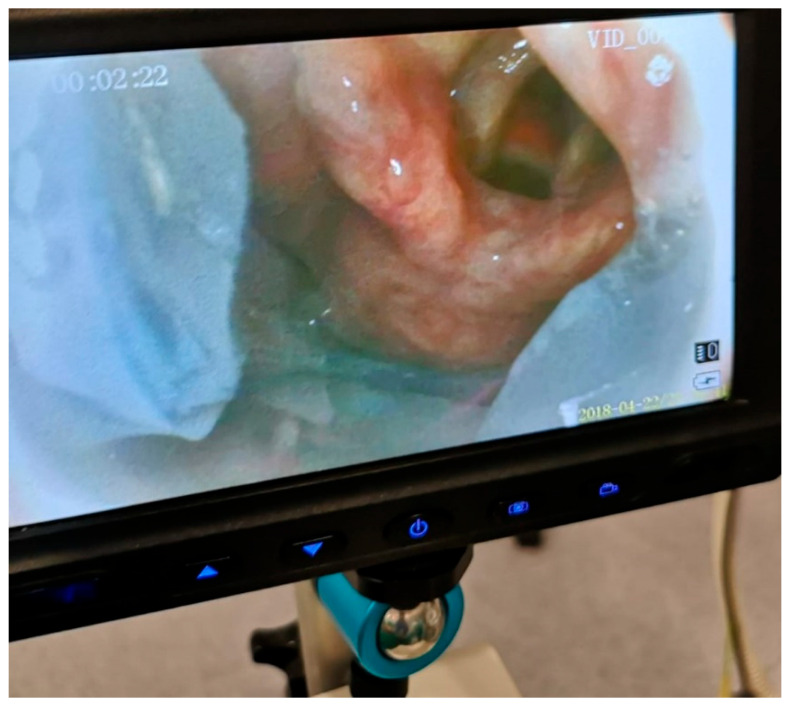
Additional monitor connected to Vision Mask video system. Vision Mask is marketed and distributed from Murcia, Spain.

It consists of five access points (Figure 5):

1. An upper left-side access point which allows measurement with a manometer to determine the pressure inside the laryngeal mask in cm H_2_O, where the pressure ranges from 10 to 20 mmHg.
2. A video stylus hole with connection. A left side access point is available for its camera stylet, which can be connected to a reusable 2.8-inch (Figure 6) and 7-inch (Figure 7) portable monitor with image/video recording capabilities. The channel is open at one end and closed at the other end, and never comes into contact with the patient.
3. Its central access allows gas inlet and outlet, plus the introduction of an endotracheal tube (ETT) for rescue intubation or fibro or a video bronchoscope, with 15 mm connection.
4. The lower right-side access has gastric access. It has an open channel at both ends. Suction catheter number 12 French or lower can be use in Vision Mask size 3 and suction catheter number 14 or lower can be use in Vision Mask size 4.
5. An upper right-side access point for a free gas outlet from the interior when using CPAP ventilation.

## 7. Advantages and Limitations of New SAD with Integrated Video System

Advantages:

- It facilitates the location of the glottis, optimising the laryngeal view.
- It offers simple insertion like any other SAD but always visualises the airway. It allows confirmation of correct placement.
- Manoeuvres can be performed to improve the anatomical view.
- It has a better anatomical fit and airway function.
- It provides confirmation that the epiglottis is seated on the outside of the device and avoids injuring it, as occurs in direct or indirect intubation, by seating the intubation device in the vallecula.
- It allows rapid diagnosis of the problem if ventilation is inadequate.
- It avoids damage and obstruction to the airway and damage to crowded airways.
- It allows intubation through the SAD of ETT under direct vision, reducing the difficulty of advancing the ETT by overcoming anatomical obstacles.
- It allows airway rescue in patients without obstructive problems due to difficulties with ventilation with face masks, poor apnoea tolerance and difficult intubation.
- Vision-guided supraglottic airway devices insertion may further eliminate a need for fibreoptic checks and videolaringoscopy checks. It also reduces the extra space needed and increased complexity for the operator when the videolaryngoscope is inserted into the oropharynx external to an SAD [56].
- It ensures that oro-pharyngeal leak pressure recordings are unaffected by spurious inaccuracy because of minor or gross SAD misalignment or incorrect positioning [56].

Limitations:

- The device has a single chamber that allows the entrance of the glottis to be seen, but the entrance of the oesophagus cannot be seen after it is in place.
- No information is obtained on the position of the distal cuff.

## 8. Conclusions

Anaesthesiology is one of the specialties that has grown exponentially. Due to technological progress, the safety of the techniques performed have improved.

In the course of history, there are milestones that are significant and revolutionary. Archie Brain revolutionised airway management by introducing the laryngeal mask into the anaesthesiologist’s armamentarium, and this device has earned its place over time. Just as US is here to stay, so too is the use of video SAD. Safety and ease are needed in everything we do, and the insertion of a SAD is far from 100% perfect if performed blindly. The insertion of a SAD with built-in video allows for a safe and effective technique. Why would we accept suboptimal positions when using an LMA if we do not accept suboptimal placement of a ETT when managing our patients’ airway? The debate will continue; we are still on the road to universal acceptance of these new devices which, in the near future, should be the gold standard.

## Figures and Tables

**Table 1:** Patient sizes and weights vs. selected Vision Mask.

Size	Patient Weight	MAX Inflation Volume	Identifying Colour
3	30–70 kg	20 mL	Green
4	70–90 kg	30 mL	Yellow
5	Coming soon	40 mL	Pink

## Data Availability

Not applicable.
